# Subendocardial Viability Ratio Predictive Value for Cardiovascular Risk in Hypertensive Patients

**DOI:** 10.3390/medicina59010024

**Published:** 2022-12-22

**Authors:** Viviana Aursulesei Onofrei, Alexandr Ceasovschih, Razvan Constantin Anghel, Mihai Roca, Dragos Traian Marius Marcu, Cristina Andreea Adam, Ovidiu Mitu, Carmen Cumpat, Florin Mitu, Adrian Crisan, Cristian Mihai Stefan Haba, Bogdan Artene

**Affiliations:** 1 “St. Spiridon” Clinical Emergency Hospital, Independence Boulevard No. 1, 700111 Iasi, Romania; 2 Department of Medical Specialties I, “Grigore T. Popa” University of Medicine and Pharmacy, University Street No. 16, 700115 Iasi, Romania; 3 Clinical Rehabilitation Hospital, Cardiovascular Rehabilitation Clinic, Pantelimon Halipa Street No. 14, 700661 Iasi, Romania; 4 Department of Management, “Alexandru Ioan Cuza” University, Blv. Carol I, 700506 Iasi, Romania

**Keywords:** hypertension, cardiovascular risk factors, arterial stiffness, Framingham score, atherosclerosis, pulse wave velocity, subendocardial viability ratio

## Abstract

*Background*: The subendocardial viability ratio (SEVR), also known as the Buckberg index, is a parameter of arterial stiffness with indirect prognostic value in assessing long-term cardiovascular risk. *Materials and Methods*: We conducted a prospective cohort study on 70 patients with uncomplicated hypertension admitted to a county medical reference hospital. We analyzed demographics, laboratory data, arterial stiffness parameters and cardiovascular risk scores (SCORE and Framingham risk scores) and aimed to identify paraclinical parameters associated with increased cardiovascular risk. *Results*: Of the arterial stiffness parameters, SEVR correlates statistically significantly with age, central and peripheral systolic blood pressure, as well as with heart rate. SEVR seems to have prognostic value among hypertensive patients by increasing the risk of major cardiovascular events assessed by SCORE and Framingham risk scores. SEVR correlates statistically significantly with serum fibrinogen (*p* = 0.02) and hemoglobin (*p* = 0.046). Between pulse wave velocity and lipid parameters (*p* = 0.021 for low-density lipoprotein cholesterol <LDL> and *p* = 0.030 for triglycerides) a statistically significant relationship was found for the study group. The augmentation index of the aorta also correlated with serum LDL-cholesterol (*p* = 0.032) and the hemoglobin levels (*p* = 0.040) of hypertensive patients. *Conclusions*: Age, abdominal circumference and Framingham score are independent predictors for SEVR in our study group, further highlighting the need for early therapeutic measures to control risk factors in this category of patients.

## 1. Introduction

Hypertension is one of the most common cardiovascular risk factors encountered, and its prevalence continues to rise in the face of primary prevention measures. Based on the fact that age and increased blood pressure values are the main determinants of arterial stiffness [1], the identification of paraclinical parameters with an active role in assessing the long-term prognosis of these patients is a research direction of the academic community in this field.

In addition to hypertension, diabetes mellitus, dyslipidemia, smoking and chronic kidney disease are involved in pathophysiological processes at the level of the vascular wall that over time lead to decreased compliance and elasticity due to changes in the extracellular matrix components at that level [2]. Arterial stiffness causes increased pulse pressure, left ventricle afterload and decreased diastolic blood pressure (BP) [3,4]. Over time, these hemodynamic changes constitute the pathophysiological basis of myocardial ischemia by increasing myocardial oxygen requirements, decreasing coronary perfusion and thus decreasing oxygen supply to the myocardium [5,6,7]. The pathophysiological implications of arterial stiffness on the development of renal dysfunction have been extensively researched, with recent data suggesting a statistically significant and independent link between the presence of arterial stiffness and reduced glomerular filtration rate in hypertensive patients [8].

The subendocardial viability ratio (SEVR), also known as the Buckberg index [9], is an arterial stiffness parameter correlated with coronary flow reserve which makes it a useful parameter in assessing coronary microvascular circulation in hypertensive patients [10,11]. Recent data from the literature attest to the existence of a gender-differentiated SEVR, with women’s risk of developing CAD being mainly secondary to an accelerated drop in aortic diastolic pressure determining myocardial perfusion impairment [12].

In the context of the continuous increase in the prevalence of cardiovascular risk factors and the continuous development of diagnostic and treatment methods, the identification of parameters with a prognostic role in this category of patients is useful in guiding therapeutic management [13].

The aim of this study was to identify SEVR’s role in the assessment of long-term cardiovascular risk in hypertensive patients as well as its determinants in the study group, with the aim of preventing potentially fatal acute cardiovascular events. We aim to use different risk scores that were validated on different populations for a comprehensive overview.

## 2. Materials and Methods

### 2.1. Study Design and Population

We conducted a prospective, single-center clinical study including 70 patients with uncomplicated hypertension, admitted to the Cardiology Department of “St. Spiridon” Hospital. Of the 70 patients, we excluded 13 patients in whom arterial stiffness parameters could not be recorded, as well as one patient with incomplete data regarding treatment or laboratory data. The final study group included 56 hypertensive patients who were followed-up for a period of 12 months.

We included patients over the age of 18 years old with a diagnosis of essential arterial hypertension and who have given their written consent to participate in the study. The exclusion criteria were the refusal of patients to participate in the study, a personal history of arrythmias (including cardiac pacing), and diagnosis of cardiac ischemic disease with-/without interventional or surgical revascularization, stroke, cancer, as well as patients in whom arterial stiffness parameters could not be assessed.

### 2.2. Measurements

We analyzed a variety of parameters such as demographics (age, gender), cardiovascular (CV) risk factors, anthropometric parameters, vital signs (systolic blood pressure <SBP, mmHg>, diastolic blood pressure <DBP, mmHg> and heart rate <beats per minute, bpm>) as well as arterial stiffness parameters. The diagnosis of hypertension has been established according to current guidelines [13].

The laboratory testing consisted of lipid profile (total cholesterol, low-density lipoprotein cholesterol, high-density lipoprotein cholesterol, tryglicerides), serum glucose, uric acid, renal function parameters (creatinine, urea), fibrinogen and hemoleucogram. The results were presented according to the International System of Units.

Cardiovascular risk scores were calculated based on the CV risk factors obtained from the medical interview, physical examination and biochemical tests. Two major CV risk scores were computed: the Framingham risk score [14,15] and SCORE (Systematic Coronary Risk Evaluation Project) risk [16,17].

### 2.3. Atherosclerosis Evaluation Using the Artheriograph Device

**Figure 1:**
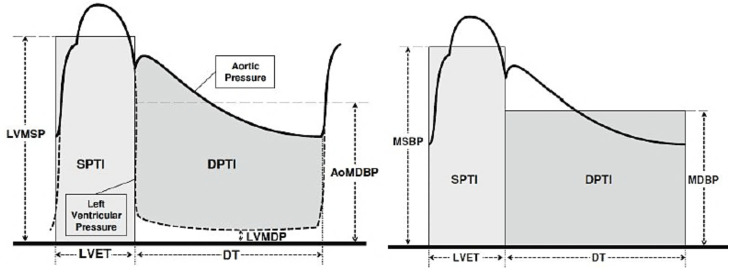
SEVR determinants (MSBP: mean blood pressure, SPTI: systolic pressure time index, DPTI: diastolic pressure time index, LVET: LV ejection duration, LVMDP: mean diastolic pressure in LV, LVMSP: mean systolic pressure in LV, DT: diastole duration) (adapted after [18]).

Using an Arteriograph, several parameters of AS were determined noninvasively, and the focus was on pulse wave velocity (PWV), the augmentation index of the aorta (AixAo), systolic area under the pulse wave curve (SAI), and the diastolic area below the pulse wave curve (DAI). SEVR was determined using the ratio of the areas of the systolic and diastolic portions below the aortic pulse wave curve and denoted as systolic area index (SAI) and diastolic area index (DAI), respectively. Knowing that SAI is calculated as the product of mean systolic LV pressure and systole duration and DAI as the product of the difference between mean aortic diastolic pressure and mean diastolic LV pressure and diastole duration, we rewrote the calculation formula as follows [18] (Figure 1):

$$SEVR=\frac{\left(mean\;aortic\;diastolic\;pressure-mean\;diastolic\;LV\;pressure\right)\;\times \;diastole\;duration\;}{mean\;systolic\;LV\;pressure\;\times \;systole\;duration\;}$$

The parameter measurement protocol via the Arteriograph device involved the following steps: (1) recording general patient data (name, date of birth, weight, height, arm circumference and abdominal circumference, the distance between the sternal notch and the upper edge of the pubic symphysis, without following the abdominal relief); (2) locating the area of maximum pulsatility of the brachial artery, with the positioning of the cuff of the device at this level; (3) the initiation of measurements, with the patient lying on his back and tracking the recording of pulse waves on the monitor to observe the morphology of the route; and (4) the interpretation of the results. In addition, before and during the recording the following measures were observed: the examination was performed in a quiet environment, and during patient mobilization, the avoidance of speech during the measurement was encouraged. When the “white coat” effect was suspected regarding hypertension, an attempt was made to reassure the patient and repeat the measurement. Smoking and coffee consumption were suppressed at least 3 h before the examination, copious meals were avoided during this period, as was the administration of nitrates. Alcohol consumption was prohibited for an period of 10 h before the administration.

### 2.4. Statistical Analysis

A statistical analysis was performed using SPSS statistics software (Statistical Package for the Social Sciences version 23 for Windows; SPSS Inc., Chicago, IL, USA). An initial descriptive analysis of the variables was performed for the continuous type variables calculating the mean, the median, minimum and maximum values, quartiles, and standard deviation. Skewness (measuring the symmetry of the variables with respect to the mean value) and kurtosis (flattening coefficient) were determined to assess the normal distribution of continuous variables by using the Shapiro–Wilk test. All numerical variables had a normal distribution and were presented as means ± standard deviation.

To compare the mean values between two groups of continuous values in order to determine the statistically significant differences, the *t*-test (independent *t* test) and ANOVA (one way analysis of variance) was used. Pearson (for continuous variables) and Spearman (for categorical variables) correlation coefficients were used to assess the presence of correlations between the studied variables. For the subsequent analysis of the relationship between the variables that met the statistical threshold for the realized correlations, a simple linear regression was performed, and as well as by selecting several independent variables that influence a dependent variable, simple linear regression was extended to multiple regression. A *p*-value < 0.05 was considered statistically significant.

### 2.5. Ethics

The study protocol was approved by the local Ethics Committee of the “Grigore T. Popa” University of Medicine and Pharmacy Iași and of “St. Spiridon” Clinical Emergency Hospital, and was conducted in accordance with the terms of the Helsinki Declaration. All participants signed an informed written consent before enrollment.

## 3. Results

We enrolled 56 patients diagnosed with uncomplicated essential hypertension who had been evaluated in an integrative and multidisciplinary approach. The study group included predominantly male patients (62.5%), with an average age of 67.62 ± 9.78 years.

In addition to demographics, the statistical analysis also included vital parameters. Table 1 lists the parameters associated with cardio-metabolic profiles according to gender. In our study, SBP had a mean value of 143.23 ± 28.81 mmHg in the whole group, with no statistically significant gender differences (*p* = 0.538). DBP had an average value of 78.96 ± 16.44 mmHg, and the mean blood pressure (MBP) averaged 100.42 ± 19.7 mmHg. Pulse pressure (PP) had had an average value of 64.26 ± 17.71 mmHg, with a mean value slightly higher in women (67.23 ± 13.81 mmHg) compared to men (62.48 ± 19.65 mmHg) (Table 1).

The mean heart rate was 67.16 ± 11.11 beats per minute (bpm) in the whole group (statistically analyzed), without identifying statistically significant differences between genders (67.33 ± 10.94 bpm vs. 67.05 ± 11.26 bpm). Regarding anthropometric parameters, special attention was paid to the value of abdominal circumference, which had an average value above the upper limit of the normal range of values for both females and males (109.88 ± 12.605 vs. 101.36 ± 11.95 cm).

In addition to demographic and hemodynamic data, the duration of hypertension was also included in the statistical analysis. Thus, three patients (7.3%) had hypertension of up to 1 year old, 10 patients (24.4%) had hypertension from 1 to 5 years since diagnosis, 12 patients (29.3%) had hypertension from 5 to 10 years since diagnosis, and 16 cases (39%) had hypertension for more than 10 years since diagnosis. In 15 patients it was not possible to determine the duration of hypertension from the medical history or existing medical documents.

We evaluated a variety of biological parameters, both hematological and biochemical, to outline the metabolic profile of the 56 patients enrolled in the study (Table 2). Thus, mean serum glucose (125.20 ± 44.35 mg/dL), low-density lipoprotein (LDL), cholesterol (121.67 ± 42.18 mg/dL) and uric acid (5.61 ± 1.62 mg/dL) levels were above the upper limit of the normal range. These parameters also represent risk factors for increased morbidity due to acute cardiovascular events in hypertensive patients who frequently associate with dyslipidemia, diabetes mellitus or changes in purine metabolism. In terms of renal function parameters, although mean serum urea and creatinine levels were within the normal range, eGFR was associated with low mean values (82.80 ± 23.89).

Regarding the parameters of arterial stiffness we used the oscillometric analysis of the pressure curves recorded at the level of the brachial artery by the Arteriograph device (Table 3). We evaluated the PWV at the central level (PWVao) whose average value was 9.75 ± 1.74 m/s, the AIx with a mean value of 32.82 ± 14.02%, the SEVR with an associated mean value of 107.87 ± 28.14%, and diastolic reflection area (DRA), with a mean value of 40.81 ± 13.22.

**Figure 2:**
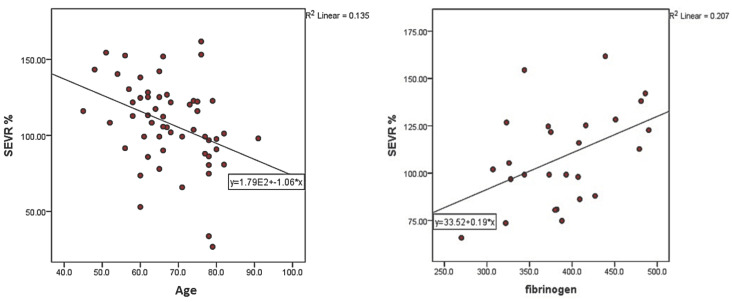
Correlation of SEVR values with age and serum fibrinogen (SEVR: subendocardial viability ratio).

**Figure 3:**
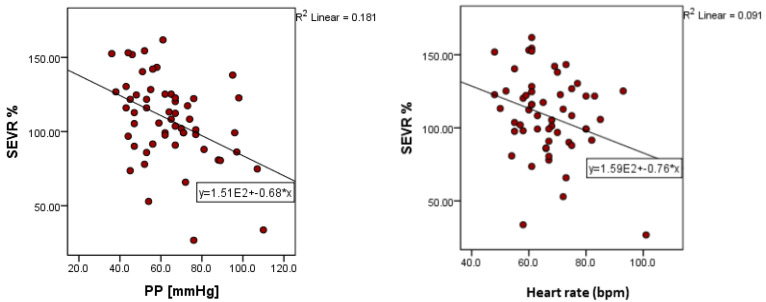
Correlation of SEVR values with pulse pressure and heart rate (SEVR: subendocardial viability ratio; PP: pulse pressure).

**Figure 4:**
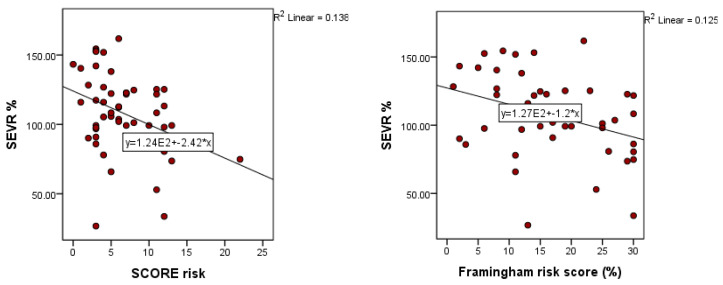
Correlation of SEVR values with SCORE risk values and Framingham risk score values (SEVR: subendocardial viability ratio).

In addition to a descriptive statistical analysis, various statistical correlations were made between SEVR and biological parameters, other arterial stiffness parameters, and risk scores as shown in Table 4 and Figure 2, Figure 3 and Figure 4. Between SEVR and age there is an inverse, statistically significant relationship (*p* = 0.005, r = −0.367) which highlights the decreasing trend of SEVR with age in our group. A simple linear regression was calculated to observe the influence of age on SEVR. A significant regression equation was highlighted (F (1.54) = 8.428, *p* = 0.005), with an R^2^ = 0.135. According to the analysis, SEVR associates a decrease of −1057 for an increase of age by one unit in the studied cases.

SEVR was also analyzed in relation to the hematological and biochemical parameters evaluated on enrolment in the study. Two statistically significant positive correlations were highlighted, with hemoglobin levels (*p* = 0.046, r = 0.27) and with fibrinogen levels (r = 0.455 at *p* = 0.02). The pathological values of fibrinogen were considered to be over 400 mg/dl, and after the application of the *t*-test there was a significant difference between patients with normal fibrinogen values (mean = 100.33 ± 23.98) and those with pathological values (mean = 119.93 ± 23.20), *t* (24) = −2.086, *p* = 0.048.

To further analyze the relationship of SEVR with fibrinogen, a simple linear regression was calculated. A significant regression equation was highlighted (F (1.24) = 6.257, *p* = 0.02), with an R^2^ = 0.207. According to the analysis, the SEVR recorded an increase of 0.193 for an increase in fibrinogen by one unit in the cases studied.

Furthermore, a series of negative correlations were observed between SEVR and central SBP (*p* = 0.023, r = −0.304), peripheral SBP (*p* = 0.008, r = −0.350), PP (*p* = 0.001, r = −0.426), and heart rate (*p* = 0.024, r = −0.301). A multiple linear stepwise regression was calculated to observe the influence of the values of the different parameters of blood pressure and HR on SEVR. A significant regression equation was highlighted (F (2.53) = 9.78, *p* < 0.001), with an R^2^ = 0.270. According to the analysis, SEVR records a decrease of −0.672 for an increase in pulse pressure by one unit, and by −0.753 for an increase in HR by one unit. Both PP (*p* < 0.01) and heart rate (*p* = 0.01) are statistically significant independent predictors of SEVR.

For the patients in the studied group, the SCORE and Framingham risk scores were also calculated. These correlated negatively with the SEVR values, for the Framingham score registering a *p* = 0.014, r = −0.353, and for the SCORE *p* = 0.007, r = −0.371. To further analyze the relationship of SEVR with SCORE, a simple linear regression was calculated. A significant regression equation was highlighted (F (1.50) = 7.995, *p* = 0.07), with an R^2^ = 0.138. According to the analysis, SEVR registers a decrease of −2.41 for an increase of SCORE by one unit. To analyze the relationship of SEVR with Framingham risk score, a simple linear regression was calculated. A significant regression equation was highlighted with an R^2^ = 0.125.

In an attempt to determine the variables that independently influence SEVR, a series of models were made by multiple linear regression. The equation with the highest value of adjusted R^2^ includes as independent predictors the abdominal circumference (β = −0.623, *p* < 0.001), age (β = −0.213, *p* = 0.031), and Framingham score (β = −0.540, *p* < 0.001), all three reaching the threshold of statistical significance (*p* < 0.05), with adjusted model R^2^ = 0.949, *p* <0.001. A significant regression equation was highlighted (F (3.8) = 69.541, *p* <0.001), with an R^2^ = 0.963. According to the analysis the SEVR decreased by −31,396 for patients with abdominal obesity, by −1513 to increase the Framingham score by one unit, and by 0.577 with the increase of age by one unit in the cases studied. Abdominal obesity, Framingham score and age are statistically significant independent predictors of SEVR.

When only continuous variables that correlated with SEVR (central SBP, PP, HR, age, fibrinogen value, Hb, Framingham and SCORE risk scores) were introduced into the analysis, a model was obtained with: SCORE (β = −0.441, *p* = 0.005), fibrinogen values (β = 0.428, *p* = 0.004), and Hb (β = 0.382, *p* = 0.013), all three reaching the threshold of statistical significance (*p* < 0.05), with R^2^ = 0.670 and adjusted R^2^ = 0.618.

A significant regression equation was highlighted (F (3.19) = 12.845, *p* < 0.001), with an R^2^ = 0.670. According to the analysis, SEVR records a decrease of −2403 for each increase in SCORE by one unit, an increase of 0.182 for an increase in fibrinogen by one unit, and an increase of 6675 with an increase in Hb by one unit in the studied cases. SCORE, fibrinogen and Hb were thus determined as statistically significant independent predictors of SEVR.

## 4. Discussion

Hypertension has a dual role as a cardiovascular risk factor and as a disease with an increasing prevalence in the context of accelerated global industrialization. It is also responsible for the occurrence of potentially fatal acute cardiovascular events in the absence of a personalized and integrative management of each individual patient. Identification of paraclinical parameters associated with increased cardiovascular risk is essential, having a dual role: prognostic and therapeutic.

In our study, we demonstrated that SERV correlates with age, fibrinogen and hemoglobin serum levels, as well as with various arterial stiffness parameters such as pulse pressure, central, and peripheral SBP.

SEVR is influenced by several factors, including demographics. In our study, we demonstrated that SEVR correlates negatively with age. In addition, based on the concept that age is an element of the Framingham score, we emphasized the impact of age on SEVR by obtaining a statistically significant correlation with this cardiovascular risk score (*p* = 0.014). SEVR modulates the long-term prognosis of patients with hypertension via associated arterial stiffness, which is an indirect marker of aging or the onset/progression of atherosclerotic processes [19]. Laugesen et al. [20] demonstrated that women with diabetes have a lower SEVR than men with (*p* < 0.01) or without diabetes (*p* < 0.001) or even women without diabetes (*p* < 0.001), with statistically significant correlations after adjusting for various cofounders such as age, BP, HR or smoking. In a similar study published recently, Kaname et al. [12] highlighted that aortic diastolic pressure decay underlies the SEVR differences between genders. Thus, women have a higher aortic diastolic pressure decay index compared to men, even after adjusting results for age, dyslipidaemia or diabetes, as well as a lower SEVR value (*p <* 0.001). Saito et al. [21] observed lower SEVR values in elderly patients with a BMI outside the normal range, increased heart rate, dyslipidaemia and increased serum glucose levels. Of the risk factors mentioned above, only age and heart rate have been shown to be independent predictors of SEVR. Changes in the vascular walls that occur with advancing age contribute to hypertension. Ma et al. [22] demonstrated through a statistical regression model that aortic and radial SEVR changes in the elderly are similar, this demographic parameter being an independent predictor in the study population [23]. Increasing arterial stiffness causes decreasing SEVR, the correlation with age being negative. The same group of researchers reported different results according to gender and age decade. Thus, while in male patients, SEVR values decreased in the third to fifth decades and then increased starting in the sixth decade, in females the increase in values up to the fifth decade was followed by a decrease starting in the sixth decade most likely secondary to the onset of menopause and its effect on arterial compliance [22,24,25]. A recently published clinical study correlates reduced SEVR and ankle-brachial index values with frequent systemic atherosclerotic disease in the elderly [26]. The different values of aortic and radial SEVR can also be explained in terms of age-associated vascular wall changes that occur more frequently in the aorta compared to peripheral arteries [27].

SEVR is an indirect parameter of the myocardial oxygen supply and demand [10]. The reduction in diastolic aortic pressure consequently causes a reduction in myocardial perfusion, thus highlighting the high susceptibility of the myocardium to various factors that infuse oxygen supply such as hypertension [28,29,30]. Between SEVR and cardiovascular risk there is an inversely proportional relationship, with decreasing SEVR values being associated with increased cardiovascular risk and worsening prognosis predominantly in patients with diabetes or chronic kidney disease [20,31,32]. Patients with chronic kidney disease and low SEVR have a high risk of myocardial oxygen demand impairment [33]. In addition to glomerular filtration rate, SEVR modulates serum cystatin C levels even in patients without kidney impairment [34].

Tsiachris et al. [10] investigated the role of SEVR as a predictor of coronary microcirculation in hypertensive patients and observed a 24.5% decrease in this parameter in hypertensive patients with low coronary flow reserve (*p* = 0.0002). The same study also highlighted the independent predictive role of age, left ventricular mass index and diastolic BP alongside SEVR for coronary flow reserve.

SEVR is an indirect marker of the pathophysiological burden of metabolic syndrome on arterial function. The pathophysiological rationale lies in the pathophysiological effect of the metabolic syndrome on subclinical vascular damage and increased arterial stiffness [35,36,37]. In our study, no statistically significant correlations were observed between SEVR and components of the lipid profile (total cholesterol and triglycerides), but clinically and prognostically significant results were recorded for abdominal circumference, which was found to be an independent predictor (*p* = 0.031). Although no statistically significant correlations were found, changes in lipid, carbohydrate and uric acid profile parameters have a similar negative impact to classical cardiovascular risk factors, contributing to the development or evolution of atherosclerotic processes, justifying changes in paraclinical parameters of arterial stiffness. Jekell et al. [31] also concluded that in hypertensive patients without diabetes mellitus, SEVR does not correlate with serum HDL-cholesterol levels or insulin resistance markers.

In analyzing our group of patients we demonstrated that patients with abdominal obesity had a 31.39 lower SEVR compared to the other patients. Our results are consistent with data presented by similar clinical studies in the literature. In a recent study, a group of investigators, Tocci et al. [38], demonstrated in a cohort of adolescents that overweight patients have reduced SEVR values (114.4 ± 25.9% vs. 132.2 ± 22.0% respectively, with a *p* value of 0.038) compared to normal-weight patients, similar to carotid-femoral pulse wave velocity and aortic systolic blood pressure (*p* = 0.043). Marčun-Varda et al. [39] analyzed a cohort of pediatric patients with different cardiovascular risk factors and observed a potential link with cardiovascular risk through correlations with age, heart rate and mean central BP, but further studies in the field are needed to confirm this. Although the value of SEVR as a predictor of decreased myocardial viability in overweight patients has not been demonstrated, its decrease secondary to consecutive myocardial work and aortic systolic pressure augmentation is a direction for future research in the field [40].

The correlation between arterial stiffness, SEVR and the cardio-metabolic risk factors have been investigated by Fantin et al. [41] in a recent study in which 55 patients with metabolic syndrome were enrolled. The group of investigators demonstrated that the presence of metabolic syndrome correlates with reduced SEVR values (*p* = 0.012), even after adjusting multivariate regression for different cofactors such as age, gender or mean arterial blood pressure (*p* = 0.040). The number of metabolic syndrome components also influences the evolution of SEVR, its values decreasing with increasing number of metabolic syndrome elements (*p* = 0.005).

Among the laboratory parameters included in the statistical analysis, between fibrinogen (*p* = 0.02), hemoglobin (*p* = 0.046) and SEVR there are statistically significant correlations for our study group. There is a complex pathophysiological relationship between anemia and cardiovascular risk, mediated in many cases by the presence of chronic kidney disease [42]. Serum blood glucose is an important cardiovascular risk factor with therapeutic and prognostic implications, although SEVR, PWV and AIx were not statistically significantly correlated with glycemia in our study group. The lack of statistically significant correlations can be explained by the normal values of the velocities obtained in the patients enrolled in the present study. Clinical studies, however, highlight the presence of altered values of arterial stiffness parameters in diabetic patients. Di Pino et al. [43] have shown that patients with prediabetes and high glomerular filtration rate values have increased augmentation pressure and AIx values and reduced SEVR values (*p* < 0.05). In patients with type 1 diabetes mellitus, reduced SEVR values correlate independently and negatively with the presence and degree of microalbuminuria and are a superior predictor of PWV in assessing the albumin excretion rate [44]. The duration of diabetes also modulates the SEVR value, which is reduced in women with type 2 diabetes diagnosed no more than 5 years ago [20].

Ekart et al. [45] demonstrated that SEVR is dependent on serum hemoglobin and troponin levels in a cohort of 91 patients with kidney disease (non-dialysis). In addition, the subgroup of patients with anemia was characterized by a higher serum creatinine level, higher blood pressure values, and a lower SEVR value than the cases without anemia. In a more recent clinical trial, the same group of investigators demonstrated that chronic kidney disease patients with an SEVR of less than 130% were associated with a 16-fold increased risk of fatal cardiovascular events compared to patients with an SEVR greater than 130% (*p* = 0.004) [46]. Not only do low eGFR values correlate with SEVR, but high ones do as well, with the main determinants of SEVR in the prediabetic population being SBP, eGFR and insulin resistance as major determinants of arterial stiffness [43]. Based on the concept that a significant percentage of hypertensive patients are associated with peripheral arterial disease, the analysis of paraclinical parameters of arterial stiffness has prognostic value. The identification of a reduced ankle-brachial index value correlates with an increased PWV value, but not with a decreased SEVR [47].

The evolution of SEVR in relation to age, oxygen saturation and serum hemoglobin level was studied in a group of 41 hospitalized heart failure patients in order to identify predictors involved in the risk of rehospitalization at 30 days [48]. Clinical improvement resulted in statistically significant improvement in SEVR interpreted both in isolation and after correcting for serum hemoglobin, leading to the conclusion that the administration of medical therapy (predominantly diuretics) induces improvement in arterial perfusion and subendocardial perfusion in geriatric patients. The variation of SEVR as a function of serum hemoglobin and arterial oxygen saturation was not only observed in hypertensive patients but also in those with orthostatic hypotension in whom SEVR values were lower (*p* = 0.05) and PWVAo higher (*p* = 0.042) [49].

In addition to demographic parameters and laboratory data, SEVR also correlates with arterial stiffness parameters. In our statistically analyzed group, we demonstrated that statistically significant correlations exist between SEVR and central SBP, peripheral SBP, and heart rate. Also, PP and frequency were found to be independent predictors of SEVR in multivariate regression, aspects correlating with the data presented in the literature. We also demonstrated that central SBP correlates statistically significantly with PWV (*p =* 0.044) and Aix (*p* = 0.029).

Anyfanti et al. [50] also emphasize the usefulness of SEVR in assessing microvascular coronary perfusion as well as its variability according to blood pressure phenotype. SEVR varies according to blood pressure phenotype, and the group of investigators observed that normotensive patients have higher SEVR values compared to those with masked hypertension, white-coat hypertension or true hypertension (*p* = 0.017). In addition, central SBP, peripheral SBP and the total arterial compliance index were found to be predictors in univariate statistical analysis, with value retained after adjusting for heart rate. Pulse pressure also influences SEVR values in elderly hypertensive patients. Chemla et al. [25] concluded that between SEVR and diastolic time over systolic time ratio there is a positive linear correlation for a given cut-off value of this ratio. SEVR is associated with lower hypertension in patients with PP over 60 mmHg compared to those with normal PP values.

Our study has several limitations due to the relatively small number of cases analyzed. We excluded patients from the study in whom arterial stiffness parameters could not be obtained or in whom the observation chart did not contain all the parameters necessary for statistical analysis.

## 5. Conclusions

Our results support the notion that the assessment of SEVR in patients with hypertension has prognostic value, being a useful pillar in the assessment of long-term cardiovascular risk by modulating SCORE and Framingham risk scores. Age, serum fibrinogen level, haemoglobin, heart rate and central and peripheral SBP are parameters that correlate statistically significantly with SEVR, but independent predictive value in multivariate statistical analysis was demonstrated only for age, abdominal circumference and Framingham risk score.

The value of SEVR as an index of long-term cardiovascular risk is even greater, as it is associated with a diversity of parameters, many of which are cardiovascular risk factors per se or have a defining role in increasing the cardiovascular risk of morbidity and mortality. These results raise the necessity of applying early specific therapeutic measures to control the CV risk factors in this group of patients.

## Figures and Tables

**Table 1:** Gender distribution of systolic blood pressure and pulse pressure.

	N	Mean	Std. Deviation	Std. Error	95% Confidence Interval for Mean Lower Bound–Upper Bound		Min	Max	*p*
**Systolic blood pressure (mmHg)**									
Females	21	145.524	26.0684	5.6886	133.658	157.390	102.0	219.0	0.538
Males	35	141.857	30.6231	5.1762	131.338	152.377	103.0	224.0	
Total	56	143.232	28.8103	3.8499	135.517	150.948	102.0	224.0	
**Pulse pressure (mmHg)**									
Females	21	67.238	13.8163	3.0150	60.949	73.527	43.0	98.0	0.305
Males	35	62.486	19.6594	3.3230	55.732	69.239	36.0	110.0	
Total	56	64.268	17.7123	2.3669	59.524	69.011	36.0	110.0	

**Table 2:** Description of the analyzed biochemical and hematological parameters.

Biological Parameters	Minimum	Maximum	Mean Value	Standard Deviation
Hemoglobin (g%)	10.30	18.00	13.5091	1.65678
Hematocrit (%)	33.00	53.10	40.4241	4.41400
Fasting glucose, mg/dL	62	287	125.20	44.357
Total cholesterol, mg/dL	105	296	195.67	45.622
HDL-cholesterol, mg/dL	14	112	46.98	15.673
LDL-cholesterol, mg/dL	55	205	121.67	42.186
Triglycerides, mg/dL	57	438	140.13	79.310
Uric acid, mg/dL	3.30	9.70	5.6116	1.62501
Fibrinogen, mg/dL	270.0	490.0	389.231	59.3743
Urea, mg/dL	16.00	75.00	40.6364	13.24465
Serum creatinine, mg/dL	0.57	1.97	0.9462	0.28369
eGFR (mL/min/1.73 m^2^)	36	135	82.80	23.896

HDL: high-density lipoprotein; LDL: low-density lipoprotein; eGFR: estimated glomerular filtration rate.

**Table 3:** Characteristics of arterial stiffness parameters.

	PWVao [m/s]	AIx Aortic [%]	SEVR %	DRA
Mean	9.757	32.823	107.8725	40.812
Median	9.750	32.050	108.3300	38.850
Standard deviation	1.7434	14.0231	28.14657	13.2270
Minimum	5.8	6.5	26.74	10.4
Maximum	14.1	63.2	161.78	75.3
Percentile				
25	8.325	23.700	91.0225	32.875
50	9.750	32.050	108.3300	38.850
75	10.900	43.800	125.1025	48.900

PWVao: pulse wave velocity at the central level; AIx: augmentation index; SEVR: subendocardial viability index; DRA: diastolic reflection area.

**Table 4:** Correlations between SEVR and hematological, biochemical parameters, or arterial stiffness parameters.

	SEVR		PWVao [m/s]		AIx Aortic [%]	
	r	*p*	r	*p*	r	*p*
**Biochemical parameters**						
Fasting glucose (mg/dL)	0.02	0.87	0.192	0.159	−0.008	0.956
Total cholesterol (mg/dL)	0.02	0.84	0.245	0.079	0.306	0.027
HDL-cholesterol (mg/dL)	0.11	0.41	−0.254	0.082	0.114	0.439
LDL-cholesterol (mg/dL)	−0.07	0.59	0.330	0.021	0.307	0.032
Triglycerides (mg/dL)	0.16	0.23	0.301	0.030	0.081	0.569
Fibrinogen (mg/dL)	0.455	0.02	0.346	0.083	−0.260	0.199
Serum urea (mg/dL)	−0.09	0.49	0.160	0.244	0.262	0.053
Serum creatinine (mg/dL)	0.04	0.77	0.014	0.917	−0.048	0.728
Uric acid (mg/dL)	−0.01	0.96	0.193	0.290	−0.107	0.561
Hemoglobin (g%)	0.270	0.046	−0.083	0.546	−0.277	0.040
Hematocrit (%)	0.211	0.125	−0.085	0.539	−0.249	0.069
**Arterial stiffness parameters**						
Central SBP (mmHg)	−0.304	0.023	0.270	0.044	0.293	0.029
Peripheral SBP (mmHg)	−0.350	0.008	0.242	0.073	−0.010	0.942
DBP (mmHg)	−0.154	0.256	0.196	0.147	−0.118	0.388
MBP (mmHg)	−0.258	0.055	0.230	0.088	−0.070	0.608
PP (mmHg)	−0.426	0.001	0.211	0.119	0.093	0.495
Heart rate, bpm	−0.301	0.024	0.203	0.133	−0.478	<0.001

r: Pearson Correlation; HDL: high-density lipoprotein; LDL: low-density lipoprotein; SBP: systolic blood pressure; DBP: diastolic blood pressure; MBP: mean blood pressure; PP: pulse pressure; bpm: beats per minute.
